# Metabolomic Derangements Are Associated with Mortality in Critically Ill Adult Patients

**DOI:** 10.1371/journal.pone.0087538

**Published:** 2014-01-30

**Authors:** Angela J. Rogers, Michael McGeachie, Rebecca M. Baron, Lee Gazourian, Jeffrey A. Haspel, Kiichi Nakahira, Laura E. Fredenburgh, Gary M. Hunninghake, Benjamin A. Raby, Michael A. Matthay, Ronny M. Otero, Vance G. Fowler, Emanuel P. Rivers, Christopher W. Woods, Stephen Kingsmore, Ray J. Langley, Augustine M. K. Choi

**Affiliations:** 1 Channing Division of Network Medicine, Brigham and Women’s Hospital, Boston, Massachusetts, United States of America; 2 Division of Pulmonary and Critical Care Medicine, Brigham and Women’s Hospital, Boston, Massachusetts, United States of America; 3 Division of Pulmonary and Critical Care Medicine, Stanford University, Stanford, California, United States of America; 4 Department of Medicine and Anesthesia, University of California San Francisco, San Francisco, California, United States of America; 5 Department of Emergency Medicine, Henry Ford Hospital, Detroit, Michigan, United States of America; 6 Duke Institute for Genome Sciences and Policy, and Department of Medicine, Duke University School of Medicine, Durham, North Carolina, United States of America; 7 National Center for Genome Resources, Santa Fe, New Mexico, United States of America; 8 Lovelace Respiratory Research Institute, Santa Fe, New Mexico, United States of America; 9 Department of Medicine, Weill Cornell Medical College, New York, New York, United States of America; Beth Israel Deaconess Medical Center, United States of America

## Abstract

**Objective:**

To identify metabolomic biomarkers predictive of Intensive Care Unit (ICU) mortality in adults.

**Rationale:**

Comprehensive metabolomic profiling of plasma at ICU admission to identify biomarkers associated with mortality has recently become feasible.

**Methods:**

We performed metabolomic profiling of plasma from 90 ICU subjects enrolled in the BWH Registry of Critical Illness (RoCI). We tested individual metabolites and a Bayesian Network of metabolites for association with 28-day mortality, using logistic regression in R, and the CGBayesNets Package in MATLAB. Both individual metabolites and the network were tested for replication in an independent cohort of 149 adults enrolled in the Community Acquired Pneumonia and Sepsis Outcome Diagnostics (CAPSOD) study.

**Results:**

We tested variable metabolites for association with 28-day mortality. In RoCI, nearly one third of metabolites differed among ICU survivors versus those who died by day 28 (N = 57 metabolites, p<.05). Associations with 28-day mortality replicated for 31 of these metabolites (with p<.05) in the CAPSOD population. Replicating metabolites included lipids (N = 14), amino acids or amino acid breakdown products (N = 12), carbohydrates (N = 1), nucleotides (N = 3), and 1 peptide. Among 31 replicated metabolites, 25 were higher in subjects who progressed to die; all 6 metabolites that are lower in those who die are lipids. We used Bayesian modeling to form a metabolomic network of 7 metabolites associated with death (gamma-glutamylphenylalanine, gamma-glutamyltyrosine, 1-arachidonoylGPC(20:4), taurochenodeoxycholate, 3-(4-hydroxyphenyl) lactate, sucrose, kynurenine). This network achieved a 91% AUC predicting 28-day mortality in RoCI, and 74% of the AUC in CAPSOD (p<.001 in both populations).

**Conclusion:**

Both individual metabolites and a metabolomic network were associated with 28-day mortality in two independent cohorts. Metabolomic profiling represents a valuable new approach for identifying novel biomarkers in critically ill patients.

## Introduction

More than 4 million patients are admitted to intensive care units (ICUs) each year in the United States. [1] Despite marked improvements in care for these critically ill patients, [2], [3] approximately 500,000 die in the ICU each year. [1] Identifying biomarkers that distinguish the patients at highest risk for poor outcomes who could be targeted for novel therapies and clinical trials is critical. [4].

Investigating metabolomics to identify novel biomarkers in the ICU is promising for several reasons. First, the most widely used biomarker of ICU mortality identified to date is lactate, a byproduct of anaerobic metabolism. Second, in the spectrum from genotype to phenotype, metabolites, like proteomics, are correlated most closely to phenotype, and are thus likely much more highly correlated with disease. [5] While metabolomics was previously highly time-intensive and technologically limited, and thus could not plausibly be done on a large scale, technological advances in gas and lipid chromatography and mass spectroscopy now make such study possible. [6], [7] Finally, metabolomic profiling has been performed in multiple complex trait diseases (notably recent work in cancer [8], diabetes [9], tuberculosis [10] and, most recently, septic shock in children [11]), in each case identifying important novel biologic pathways that contribute to pathogenesis and prognosis.

For all of these reasons, we performed metabolic profiling to identify prognosticators of 28-day mortality in a cohort of 90 critically ill adult ICU patients. We identified both widespread differences in individual metabolites and a network of interacting metabolites associated with 28-day mortality. The association of these metabolites with 28-day mortality was then replicated in an independent cohort of 150 adults with sepsis. We then compare our results to recent work that examined metabolomics in septic shock using these same metabolomics profiles but different analytical methods. [12].

## Methods

### Populations

#### Brigham and Women’s Hospital (BWH) Registry of Critical Illness (RoCI)

The BWH RoCI is approved by the Partners IRB committee (2008-P-000495). The protocol for recruitment for RoCI has been published in detail elsewhere. [13] Briefly, adult patients admitted to the BWH Medical Intensive Care Unit are eligible for inclusion in the RoCI within 72 hours of presentation, unless certain exclusion criteria are met (see **Methods S1**). Plasma is obtained on days 1, 3, and 7 of enrollment. Extensive phenotypic data (including age, gender, key comorbidities, and APACHE II score), laboratory, radiologic, and mortality data are recorded for all subjects. Between September 2008 and May 2010, 225 subjects were enrolled in the RoCI. Among these 225 subjects, 90 subjects were selected for metabolic profiling: 29 with SIRS, 30 with Sepsis, and 31 with sepsis-induced ARDS. Cases were selected for profiling based in part on IL-18 levels as part of a separate analysis [13] (sepsis and SIRS patients with low IL-18 levels, ARDS with high IL-18 levels; 31 of the 34 ARDS cases at that time were used). Cases were not selected with regards to risk of death or any known metabolic feature.

#### CAPSOD population

The protocol for enrollment in the Community Acquired Pneumonia and Sepsis Outcome Diagnostics (CAPSOD) study has been previously published. [12], [14], [15] Briefly, 1152 patients with sepsis (≥2 Systemic Inflammatory Response (SIRS) criteria and presumed infection) were enrolled in emergency rooms associated with 3 US Hospitals. Blood samples and extensive phenotypic and laboratory data were recorded at enrollment. Survival/death was the primary outcome. The validation set of 149 patients (13% of the total CAPSOD cohort) was selected in five groups that reflected conventional concepts of sepsis progression as a pyramid from SIRS to sepsis and septic shock (see **Methods S1**).

### Metabolomic Profiling

Metabolomic profiles were generated independently for the BWH RoCI samples (N = 90 plasma samples from Day 1 of enrollment (within 72 hours of ICU admission), targeting 411 metabolites) and the CAPSOD samples (N = 150 targeting 439 metabolites) by Metabolon, Inc. Gas and liquid chromatography and mass spectroscopy was performed as described previously [16], [17] and in the **Methods S1**. We removed metabolites with the lowest IQR of variability in the RoCI data, leaving 308 metabolites. We then limited statistical analyses to the metabolites that were also profiled in the CAPSOD database (N = 167 identical metabolites).

### Statistical Analysis

#### Logistic regression

All raw metabolite concentrations were log_2_ transformed and normalized. We performed logistic regression in Rv.2.14, after adjustment for age, gender, race, malignancy status, and renal function (as estimated by the GFR-MDRD). Metabolites significantly associated with mortality in the RoCI cohort (p<.05) were tested for replication in CAPSOD; the significance level of p<.05 (one-sided) was used to define replication in the CAPSOD cohort.

#### Bayesian networks

Comprehensive details of Bayesian network selection are available in the **Methods S1**. Briefly, metabolite levels were log_2_ transformed and normalized separately in the RoCI and CAPSOD datasets. We then used 5-fold cross-validation on RoCI to arrive at hyper parameters for the Bayesian likelihood calculations. We performed 2500 bootstrap realizations of the RoCI dataset, and learned a pheno-centric conditional Gaussian Bayesian network [18] for each bootstrap realization. From the sample of 2500 networks, we built a consensus network by starting with the phenotype node and then adding, in sequence, the most frequent edge occurring in the bootstrap networks, and measuring the performance of that network on the dataset in cross-validation to estimate value of adding additional nodes to the network. Demographic variables were eligible for inclusion in the model, but none were ultimately selected. The network was then tested for association in CAPSOD without any parameter refitting. Predictive performance of the network was assessed by the convex hull of the Area Under the Receiver Operator Characteristic Curve (AUC). [19] Statistical significance was determined using the method of DeLong et al. [20] All Bayesian and related statistical computations were performed using the CGBayesNets package [21], [22] in MATLAB. To compare our metabolomic network to that identified in a recent manuscript derived in CAPSOD and replicated in RoCI using the same metabolomics profiling data [12], we constructed a Bayesian network comprised of their variables and compared the predictive performance using the CGBayesNets package described above. Please see the **Methods S1** for more details.

## Results

Baseline characteristics of subjects participating in the RoCI and CAPSOD cohorts are shown in **Table 1**. RoCI subjects were recruited after admission from the ICU, while CAPSOD subjects were enrolled in the ED with suspected sepsis. Of 149 CAPSOD subjects, 65 were admitted directly to the ICU on the day of admission and metabolomic profiling. As noted, RoCI subjects were younger, more frequently had cancer, had a higher baseline APACHE II score, were more often Caucasian, and had a higher mortality rate (all p<.05 by Wilcoxson or Fisher’s exact tests, **Table 1**). Of the 90 RoCI subjects, 60 had sepsis, including 25 of the 30 patients who died. As shown in Table 1, patients who die in the ICU differ in several phenotypic ways from those who survive, including higher APACHE score, more malignancy, and older age, among other variables.

**Table 1:** Baseline Characteristics.

	RoCI (N = 90				CAPSOD (N = 149)			Roci vs CAPSOD
	Live (N = 60)	Die (N = 30)		Pval	Live (N = 115)	Die (N = 34)	Pval	Pval
Age	53 (14)		58 (15)	0.15	58 (17)	69 (16)	0.002	0.009
Apache Score	23 (9)		30 (11)	0.00	15 (7)	23 (8)	8.90E–06	1.92E-11
MDRD_GFR	67 (60)		59 (43)	0.60	62 (57)	51 (40)	0.356	0.364
Days Blood Draw	2.2 (1)		2.1 (1)	0.42	0 (0)	0 (0)	N/A	1.03E-49
Male gender	28 (47%)		11 (37%)	0.50	53 (46%)	15 (44%)	1.000	0.789
Malignancy	15 (25%)		20 (67%)	0.00	10 (9%)	9 (26%)	0.015	5.25E-06
White Race	42 (70%)		28 (93%)	0.01	35 (30%)	7 (21%)	0.288	5.35E-14
Chronic Kidney Disease	10 (17%)		9 (30%)	0.17	26 (23%)	9 (26%)	0.731	0.844
Immunosuppressive Meds	14 (23%)		16 (53%)	0.01	6 (5%)	2 (6%)	1.000	2.04E-08
Diabetes	18 (30%)		3 (10%)	0.04	38 (33%)	13 (38%)	0.681	0.082

Shown are mean (SD) or N (%). P values are based on Wilcoxson or Fisher’s exact test as appropriate.

### RoCI Initial Cohort

We first assessed each of the 187 metabolites measured in both cohorts for an association with 28-day mortality. Nearly one third of the metabolites (N = 57) were associated with 28-day mortality in the RoCI cohort, after adjusting for age, gender, race, cancer status, and renal function (p<.05, **Table S1**). While p values were attenuated, the majority of metabolites remain significantly associated with death, even after further adjustment for APACHE II score, diabetic status, and use of immunosuppressive medication, including chemotherapy or systemic corticosteroids (**Table S1**). A majority of the 57 metabolites associated with death in the entire cohort were significant even when limited to the 55 RoCI patients without cancer (N = 35 of the 57 metabolites, p<.05, **Table S1**).

### Replication Cohort

**Figure 1:**
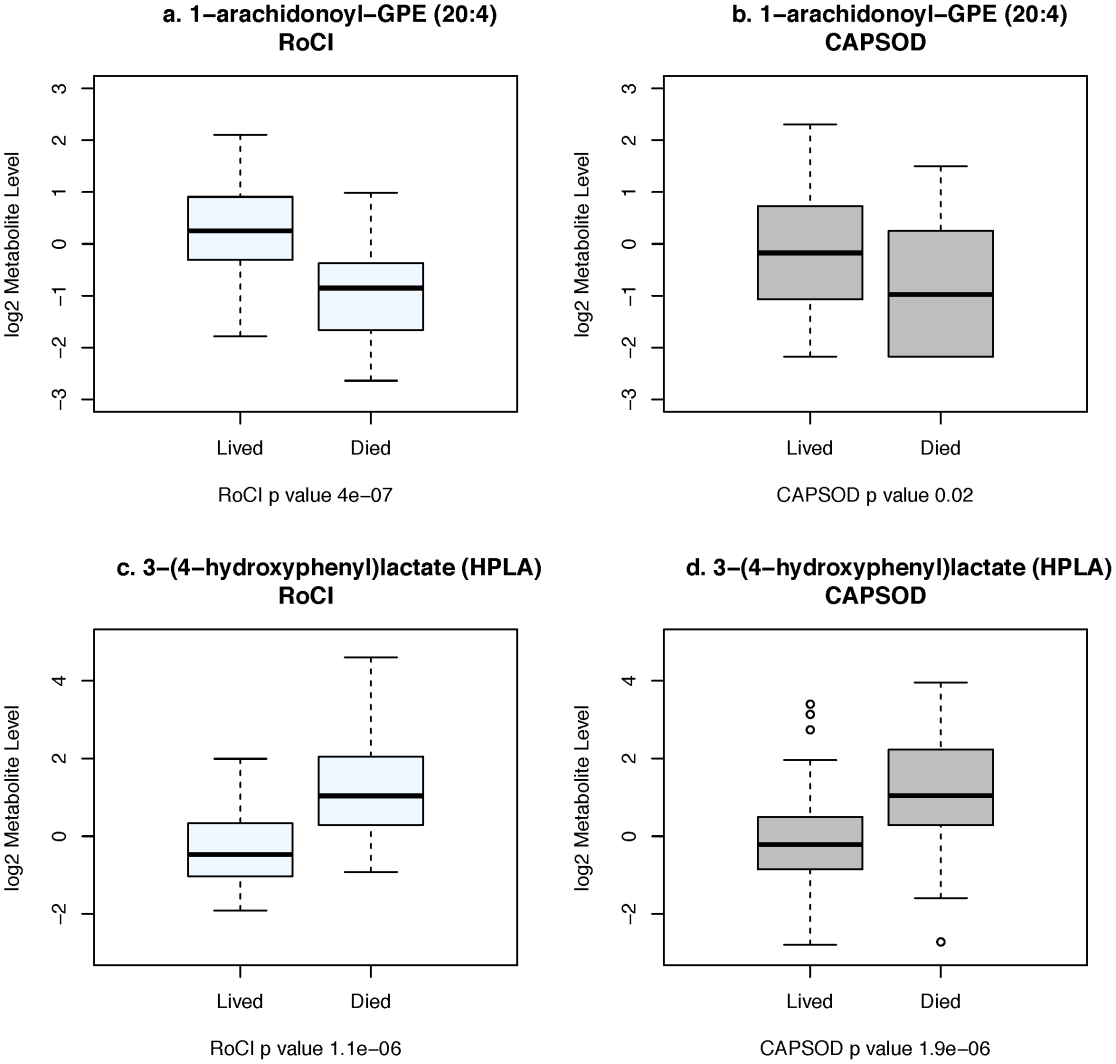
Levels of 1-arachidonoylglycero-phosphoethanolamine and sucrose are associated with 28-day mortality in both RoCI and CAPSOD cohorts. Log_2_-normalized level of 1-arachidonoylGPE(20:4), the most significantly associated metabolite in the RoCI cohort, is higher among ICU survivors in both the RoCI (light) and CAPSOD (dark) cohorts (Figure 1A and 1B). 3-(4-hydroxyphenyl) lactate (HPLA) is lower among ICU survivors in both cohorts (Figure 1C and 1D). Boxplots depict median (line) and range (edges of boxplots). P values are based on Wilcoxon’s test and are 1-sided in CAPSOD.

We then tested the 57 metabolites for replication in the CAPSOD cohort. Among the 57 metabolites associated with death in RoCI, 31 replicated in this independent population (again, after adjustment for gender, age, race, malignancy status, and renal function, p_1-sided_<.05). Replication of 31 metabolites is greater than the ∼3 replications expected by chance (p_Fisher’s exact_ = 6×10^−9^). Top metabolites are shown in **Table 2**, with a comprehensive list in **Table S2**. As shown, elevated lactate level was significantly associated with death in both populations, though it was not the top predictor. Plasma level of 1-arachidonoylglycero-phosphoethanolamine, the most significantly associated metabolite in RoCI, is shown in **Figure 1a and 1b**, with lower levels associated with mortality in both cohorts. Conversely, higher levels of 3-(4-hydroxyphenyl) lactate, a product of tyrosine and phenylalanine catabolism are associated with mortality in both cohorts (**Figure 1c and 1d**). Replicating metabolites cross multiple metabolic pathways, and include lipids (N = 14) amino acids and their breakdown products (N = 12), carbohydrates (N = 1), nucleotides (N = 3), and one peptide.

**Table 2:** Top 20 replicated metabolites, logistic regression.

Metabolite	Class	RoCI β	Roci P value	CAPSOD β	CAPSODP value
1-arachidonoyl-GPE (20:4)	Lipid	−1.51	0.0001	−0.41	0.0142
3-(4-hydroxyphenyl) lactate (HPLA)	Amino acid	1.09	0.0003	0.86	1.3E−05
5taurochenodeoxycholate	Lipid	0.59	0.0007	0.22	0.0028
taurocholate	Lipid	0.48	0.0015	0.21	0.0077
gamma-glutamylphenylalanine	Peptide	1.59	0.0017	0.61	0.0272
glycochenodeoxycholate	Lipid	0.58	0.0020	0.19	0.0114
1-arachidonoyl-GPC (20:4)	Lipid	−0.56	0.0032	−0.30	0.0016
glycocholate	Lipid	0.53	0.0036	0.18	0.0259
Hydroxyisovaleroyl-carnitine (C5)	Amino acid	0.79	0.0041	0.40	0.0317
hexanoylcarnitine (C6)	Lipid	1.15	0.0053	0.97	6.6E−05
lactate	Carbohydrate	1.11	0.0071	0.69	0.0084
alpha-hydroxyisovalerate	Amino acid	0.72	0.0071	0.58	3.8E−04
1-methylimidazoleacetate	Amino acid	0.63	0.0082	0.38	0.0104
isobutyrylcarnitine (C4)	Amino acid	0.71	0.0095	0.34	0.034
beta-hydroxyisovalerate	Amino acid	0.83	0.0134	0.37	0.0364
kynurenate	Amino acid	0.46	0.0137	0.37	0.0494
2-methylbutyroylcarnitine (C5)	Amino acid	0.75	0.0142	0.59	0.0024
1-linoleoyl-GPC (18:2)	Lipid	−0.42	0.0155	−0.30	0.0162
propionylcarnitine (C3)	Lipid	0.90	0.0156	0.62	0.0013
cortisol	Lipid	0.62	0.0175	0.56	0.0118

β and p values shown are for results for association with 28-day mortality, using logistic regression, after adjustment for age, gender, race, malignancy status, and GFR. CAPSOD p values are 1-sided, only metabolites with consistent direction were considered replication. Metabolite levels were log_2_ transformed for analysis. Data for all 31 replicated metabolites are shown in **Supplemental Table S2**.

Among the 31 metabolites associated with mortality in both cohorts, the vast majority (25) were higher in patients who died. This result is much higher than the ∼15 one would expect by chance (p_Fisher’s exact_ = .02). All 6 metabolites that were lower in non-survivors were part of the lipid metabolism pathway (**Table S2**), though lipid metabolites represented only a minority of metabolites studied (76 of the 187).

### A Metabolomic Network Associated with 28-day Mortality

**Figure 2:**
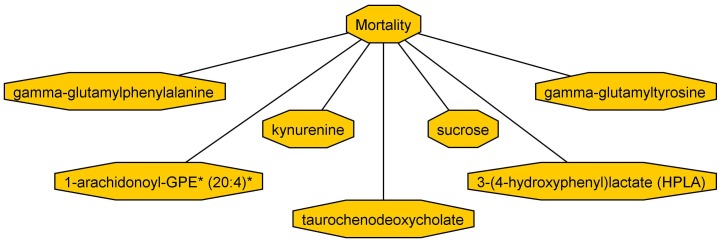
Relationship of 5 metabolites included in Bayesian Network to 28-day mortality. This network maximizes the posterior likelihood of data over all networks predictive of mortality. This network achieves 91% AUC in RoCI and 74% AUC in CAPSOD. Directed edges indicate statistical dependence and do not represent causative links.

Given the substantial proportion of metabolites that were altered in ICU survivors vs. non-survivors, we hypothesized that developing a predictive network of interacting metabolites would facilitate identification of individuals at high risk of death. We used a Bayesian network approach to identify metabolic networks, developing Bayesian networks in 2500 bootstrap realizations of the RoCI cohort and identifying those metabolites that appeared in the largest number of networks. No clinical variables were included in a sufficient number of networks to be included in the final model. Results of this iterative Bayesian Network approach are shown in **Figure 2**. This network contains seven metabolites, including two lipids, 2 amino acid/amino-acid break-down derivatives, 2 peptides, and 1 carbohydrate (kynurenine, sucrose, gamma-glutamylphenylalanine, gamma-glutamyltyrosine, 1-arachidonoyl-GPC (20:4), taurochenodeoxycholate, 3-(4-hydroxyphenyl) lactate) to predict 28-day mortality. Interestingly, the Bayesian network overlaps substantially with the top metabolites selected with logistic regression (all 7 are among the top 12 metabolites in Roci, **Table S1**).

To evaluate the performance of this network, we tested the AUC for death in both the BWH and CAPSOD cohorts. The network achieved an AUC for 28-day mortality of 91% in BWH and 74% in CAPSOD. Both of these numbers represent statistically significant prediction (p<0.001 in both cohorts).

### Comparison of Our Model with Prior Metabolomics Analyses in these Cohorts

These same cohorts and metabolomics profiles were previously analyzed, using the CAPSOD cohort as discovery and RoCI as replication, to identify a metabolomics profile associated with sepsis-related mortality. [12] Using different analytical methods, their final model included 5 metabolites (cis-4-decenoylcarnitite, 2-methylbytyroylcarnitine, butyroylcarnitine, hexanoylcarnitine, lactate, age, and hematocrit) and two phenotypic variables, hematocrit and age. Notably, none of the 5 metabolites were selected in our final model, although 4 of the metabolites were significant by logistic regression in RoCI (all but cis-4-decenoylcarnitite, **Table 2**). When the 5 metabolites and 2 phenotypes were combined into a Bayesian Network model (with Bayes Factors optimized in CAPSOD, and then tested without refitting in the RoCI cohort), the model performed similarly to our 7-metabolite model, explaining 84% of the AUC for death in CAPSOD and 84% in RoCI (p = .06 and.23 for comparisons with our models in CAPSOD and RoCI, respectively).

## Discussion

In this analysis, we tested 187 metabolites in two distinct cohorts of acutely ill patients to assess the potential for metabolomic profiling to identify biomarkers associated with death. The most important findings can be summarized as follows. First, in both populations, a substantial proportion (approximately one-third of all metabolites evaluated) were different in subjects who lived or died with a p<.05; this is a much greater number than would be expected by chance (∼8), and suggests major derangements in metabolism that are present within 24–72 hours of ICU admission in these patients. There were 31 individual metabolites associated with 28-day mortality in both cohorts; these metabolites were not restricted to a given metabolic pathway, but instead included diverse lipid, carbohydrate, amino acid, and nucleotide products. In addition to the analysis of individual metabolites, we developed a network model using a novel Bayesian Network technique that similarly replicated in both populations, explaining 91% of the AUC of death in the RoCI cohort, and 74% of the AUC in CAPSOD.

It is important to evaluate our analyses in the context of two recent metabolomics studies in critical illness, those of Mickiewicz et al. [11] in septic shock, and Langley et al., [12] who analyzed the same two adult cohorts described in this work, focusing on septic shock mortality. Mickiewicz et al. tested 57 metabolites and identified a network of 18 metabolites that were associated with mortality in a single pediatric population with septic shock using principal components analysis. Several of our top metabolites were not studied. Metabolomics may enable identification of important differences in the pathophysiology of sepsis in adults versus children, but further work, including inclusion of identical metabolites, is needed. Langley et al. approached our same data sets, but tested first in the CAPSOD population, replicating in RoCI, and using different analytical methods to identify a predictive network. It is interesting to note that, even using the same metabolomics datasets, differences in analytic strategies led to completely different networks identified, without a single common metabolite in the 2 networks, yet both highly predictive of 28 day mortality in both cohorts. This difference highlights the fact that it is probably premature to focus on a single metabolite or two, or even a single network, as several metabolites have the potential to uncover novel biology. The fact that the derangements are so widespread (with nearly 1/3 of metabolites differing in ICU survivors vs. those who die) is itself noteworthy.

Several aspects of this analysis are worth highlighting. First, metabolomic profiling in critically ill adults to identify risk metabolites is only now feasible because of advances in mass spectroscopy and lipid chromatography. Second, while the RoCI cohort was recruited within the ICU, the CAPSOD replication cohort was comprised of ED patients with presumed sepsis or community acquired pneumonia. The ED is a critical site for identifying patients at highest risk for mortality [23], and biomarkers are particularly useful for patient triage or potential enrollment in clinical trials early in the course of illness. Thus, the 31 metabolites associated with mortality in both RoCI and CAPSOD may be especially promising biomarkers. Third, we used Bayesian networks to identify interdependent metabolites of interest that together are associated with increased risk of death. Bayesian networks are an attractive modeling methodology since they can model complex interactions between many variables of interest. [24] Finally, to our knowledge, this study is the first to use Bayesian networks not only to identify novel metabolomic biomarkers, but to test the predictive ability of a network of metabolites measured in an ICU setting to predict 28-day mortality in an independent replication cohort. A replicated Bayesian network is an important strength of this study, because it allows the network to be used to construct a straightforward “ICU mortality prediction score.”

Several features of the metabolomic results in our study are particularly interesting. Among the 31 metabolites that showed replicated association with mortality, the vast majority (25) were higher among those who died in the ICU. Interestingly, all 6 metabolites that were lower among subjects who died were part of the lipid metabolism pathway (**Table S2**). Mechanisms for this finding are unclear, but merit future functional validation. Several variables known to be associated with ICU mortality were also identified in our data, adding to the validity of our findings, First, lactate levels were higher in those who died in both RoCI and CAPSOD (p = .003 and.009, respectively, **Table 2**). Similarly, cortisol has long been recognized to be elevated in critically ill patients because of hypothalamus-pituitary-adrenal axis stimulation, [25], [26] though exact mechanisms for cortisol elevation are still being elucidated [27]. As expected, free cortisol level was lower among ICU survivors in both the RoCI and CAPSOD populations (p = .02 in RoCI,.01 in CAPSOD, **Table S1**).

The 187 metabolites examined in our analysis represent a small fraction of the more than 5000 human metabolites in plasma. In addition to the 187 metabolites in this analysis, an additional 121 metabolites were measured and variable in the RoCI cohort. We focused only on metabolites that could be replicated in CAPSOD; however, as shown in **Table S3**, an additional 36 metabolites are nominally associated in RoCI (p<.05), highlighting the existence of numerous additional metabolomic differences among survivors of critical illness.

Our work has several limitations. First, each of the 7 metabolites incorporated in our model was chosen because it was selected in the largest number of models created during iterative bootstrapping. While the resulting model replicated in the independent CAPSOD cohort, reinforcing its potential importance, it is highly probable that other metabolites contribute to these results and were missed. Determining the functional role of these metabolites will require laboratory-based experimental follow-up. We studied plasma of patients early in the course of critical illness; metabolomic changes on a cellular or organ-specific nature (like those identified with glutamine uptake and cancer [8], for example) could well be missed in a plasma sample. The RoCI subjects were enrolled in the ICU, a median of 2 days after ICU admission; the CAPSOD cohort we studied was at time 0 in the emergency room. Thus, our analytic strategy favored metabolites that remain altered over days in the ICU, and may have missed metabolites with biomarker potential that change more rapidly. Also RoCI subjects derived from a medical ICU, and mortality in the cohort is heavily driven by sepsis; similarly a majority of CAPSOD subjects had infection at enrollment. This may limit the generalizability of the results of this study in a non-medical ICU context. Nutritional status can substantially alter the plasma metabolomic profile; future studies will be strengthened by including dietary information as a covariate. [28], [29] Because we were focused on discovering novel biomarkers and the overall differences in metabolomics profiling in the ICU, we chose a liberal α threshold of.05 in our logistic regression analyses in the RoCI discovery cohort. While we mitigated our risk of false positives by focusing only on replicated metabolites, it is certainly possible that false positives remain. Finally, the two cohorts differ in many baseline covariates, as shown in **Table 1**. While such heterogeneity likely means that some important metabolites did not replicate (if they are, for example, more important in cancer patients or vary with race), it also suggests that the identified metabolites are highly generalizable, and could be important in a wide variety of settings.

In summary, the combination of metabolomic profiling and Bayesian network analysis identifies both individual metabolites and a metabolomic network that are present in plasma early in the course of critical illness in adults and are associated with higher 28-day mortality in two different cohorts. Metabolomic profiling may provide new insights into both pathogenesis and prognosis in critically ill patients. Conceivably, better risk stratification for clinical trials and therapeutics can be achieved, perhaps by combining metabolomics measures with known protein biomarkers and standard clinical predictors.

## Supporting Information

Table S1 **Comprehensive Results, 187 Metabolites in RoCI.** ^1^ P value and β are for association of a given metabolite with 28-day mortality in the RoCI cohort, using logistic regression after adjustment for age, gender, race, and renal function. Metabolite values are log_2_-transformed for testing. ^2^ P value and β are for association of a given metabolite with 28-day mortality in the RoCI cohort, using logistic regression after adjustment for all variables as described above (^1^), but additionally adjusted for baseline APACHE score. ^3^ P value and β are for association of a given metabolite with 28-day mortality in the RoCI cohort, using logistic regression after adjustment for all variables as described above (^2^), but additionally adjusted for DM status and use of immunosuppressive medications, including chemotherapy and/or systemic corticosteroids. ^4^ P value and β are for association of a given metabolite with 28-day mortality in the RoCI cohort using logistic regression after adjustment for all variables as described above (^1^), but limited to the 55 subjects without malignancy, of whom 10 died.
(PDF)

Table S2 **31 metabolites associated with mortality in both cohorts.** ^1^Metabolite class is defined by Metabolon, inc. ^2^P value and β are for association of a given metabolite with 28-day mortality in the RoCI cohort, using logistic regression after adjustment for age, gender, race, malignancy status, and renal function. Metabolite values are log_2_-transformed for testing. ^3^P value in CAPSOD is similarly for logistic regression after the same adjustments. P values are 1-sided, as only metabolites with consistent direction are considered replicated.
(PDF)

Table S3 **Association with mortality in all 308 Metabolites tested in RoCI.** Y: Metabolite tested only in RoCI, not in CAPSOD. P values and β are for association of a given metabolite with 28-day mortality in the RoCI cohort, using logistic regression after adjustment for age, gender, race, malignancy status, and renal function. Metabolite values are log_2_-transformed for testing.
(PDF)

Methods S1 (PDF)
