# Supplementary material for: Metabolomic Derangements Are Associated with Mortality in Critically Ill Adult Patients
Source: PLoS One. 2014 Jan 30;9(1):e87538. doi: 10.1371/journal.pone.0087538 (PMC3907548; doi:10.1371/journal.pone.0087538)
Supplement: Table S1 — Comprehensive Results, 187 Metabolites in RoCI. 1 P value and β are for association of a given metabolite with 28-day mortality in the RoCI cohort, using logistic regression after adjustment for age, gender, race, and renal function. Metabolite values are log2-transformed for testing. 2 P value and β are for association of a given metabolite with 28-day mortality in the RoCI cohort, using logistic regression after adjustment for all variables as described above (1), but additionally adjusted for baseline APACHE score. 3 P value and β are for association of a given metabolite with 28-day mortality in the RoCI cohort, using logistic regression after adjustment for all variables as described above (2), but additionally adjusted for DM status and use of immunosuppressive medications, including chemotherapy and/or systemic corticosteroids. 4 P value and β are for association of a given metabolite with 28-day mortality in the RoCI cohort using logistic regression after adjustment for all variables as described above (1), but limited to the 55 subjects without malignancy, of whom 10 died. (PDF) [file pone.0087538.s001.pdf]

Supplemental Table S1. Comprehensive Results, 187 Metabolites in RoCI

Metabolite	β Mort[1]	P value Mort[1]	β, adj APACHE[2]	P val, adj. Apache[2]	β, adj APACHE, DM, IS[3]	Pval adj APACHE, DM, IS[3]	β Mort, non-CA (N=55)[4]	Pval, non-CA (N=55)[4]
1-arachidonoyl-GPE (20:4)	-1.51	1.00E-04	-1.44	3.00E-04	-1.36	8.00E-04	-1.71	0.003
3-(4-hydroxyphenyl)lactate (HPLA)	1.09	3.00E-04	1.02	8.00E-04	0.97	0.0022	1.64	0.0036
gamma-glutamyltyrosine	2.06	5.00E-04	1.99	9.00E-04	1.96	0.0012	4.22	0.0031
taurochenodeoxycholate	0.59	7.00E-04	0.56	0.0014	0.55	0.0031	0.61	0.011
kynurenine	1.54	0.0012	1.51	0.0044	1.38	0.0089	4.69	0.0145
sucrose	0.35	0.0014	0.32	0.0037	0.34	0.0038	0.37	0.0149
taurocholate	0.48	0.0015	0.45	0.0032	0.44	0.0055	0.52	0.0208
gamma-glutamylphenylalanine	1.59	0.0017	1.47	0.0037	1.51	0.005	2.72	0.0099
glycochenodeoxycholate	0.58	0.002	0.57	0.0027	0.57	0.0036	0.77	0.0212
indolelactate	0.95	0.0024	0.86	0.0063	0.8	0.0102	1.05	0.023
1-arachidonoyl-GPC (20:4)	-0.56	0.0032	-0.51	0.0111	-0.48	0.0192	-0.53	0.0227
1-oleoyl-GPE (18:1)	-1.05	0.0032	-0.99	0.0058	-0.92	0.0104	-0.83	0.0718
glycocholenate sulfate	0.63	0.0033	0.58	0.0099	0.54	0.0153	0.86	0.0156
glycocholate	0.53	0.0036	0.52	0.0058	0.58	0.0056	0.51	0.0587
mannose	-1.36	0.004	-1.73	0.0023	-1.6	0.0053	-1.69	0.0381
hydroxyisovaleroylcarnitine (C5)	0.79	0.0041	0.72	0.0105	0.7	0.0163	1.33	0.0189
stearidonate (18:4n3)	-0.88	0.0043	-0.89	0.0051	-0.91	0.0064	-1.1	0.0115
hexanoylcarnitine (C6)	1.15	0.0053	1.02	0.011	0.98	0.02	1.86	0.0082
isovalerylcarnitine (C5)	0.82	0.006	0.74	0.0127	0.7	0.0242	2.14	0.0052
lactate	1.11	0.0071	1.14	0.0077	1.21	0.01	1.18	0.0308
alpha-hydroxyisovalerate	0.72	0.0071	0.87	0.0035	0.85	0.0076	0.77	0.0321
1-methylimidazoleacetate	0.63	0.0082	0.56	0.0222	0.5	0.0437	0.69	0.016
glycolithocholate sulfate	0.39	0.0095	0.38	0.0148	0.44	0.0083	0.36	0.0724
isobutyrylcarnitine (C4)	0.71	0.0095	0.63	0.0273	0.67	0.025	1.04	0.01
taurocholenate sulfate	0.42	0.0101	0.37	0.0227	0.31	0.064	0.56	0.0213
biliverdin	0.72	0.013	0.73	0.0157	0.74	0.023	1.11	0.0195
beta-hydroxyisovalerate	0.83	0.0134	0.8	0.0165	0.75	0.0296	0.66	0.1713
kynurenate	0.46	0.0137	0.4	0.0656	0.38	0.0783	0.68	0.0244
2-methylbutyroylcarnitine (C5)	0.75	0.0142	0.65	0.0398	0.56	0.0785	1.39	0.0078
dihomolinolenate (20:3n3 or 3n6)	-1.23	0.0151	-1.14	0.0307	-0.96	0.0744	-1.77	0.0201
1-linoleoyl-GPC (18:2)	-0.42	0.0155	-0.36	0.0485	-0.37	0.0553	-0.42	0.0728
propionylcarnitine (C3)	0.9	0.0156	0.85	0.0228	0.76	0.0421	3	0.0036
cortisol	0.62	0.0175	0.55	0.0366	0.69	0.0176	1.73	0.0055
allantoin	0.52	0.0191	0.43	0.066	0.42	0.0735	0.76	0.0204
N2,N2-dimethylguanosine	0.67	0.0213	0.55	0.0864	0.54	0.094	0.6	0.1178
1-oleoyl-GPC (18:1)	-0.43	0.0216	-0.37	0.062	-0.37	0.0716	-0.39	0.1103
tiglyl carnitine (C5)	0.7	0.0219	0.58	0.069	0.51	0.1137	0.96	0.023

xanthine	0.51	0.022	0.61	0.0152	0.56	0.0277	0.69	0.0375
N-acetylalanine	1.32	0.0243	1.1	0.0718	0.98	0.1116	1.49	0.0609
N-acetylserine	0.71	0.0263	0.58	0.1035	0.57	0.11	1	0.0388
taurolithocholate 3-sulfate	0.33	0.027	0.31	0.0392	0.3	0.0457	0.39	0.0576
methionine	0.96	0.029	0.94	0.0332	0.87	0.0528	1.72	0.012
butyrylcarnitine (C4)	0.81	0.0306	0.82	0.0309	0.78	0.0502	1.04	0.0529
erythronate	0.59	0.0306	0.47	0.1236	0.48	0.1195	0.66	0.0756
glucose	-1.23	0.0315	-1.44	0.0251	-1.21	0.073	0.05	0.9472
glucuronate	0.36	0.0333	0.28	0.1418	0.28	0.1557	0.42	0.0754
bilirubin	0.38	0.0348	0.41	0.0266	0.44	0.026	0.35	0.1675
ornithine	0.51	0.0361	0.46	0.0545	0.41	0.0886	0.52	0.1496
2-palmitoyl-GPC (16:0)	-0.42	0.0365	-0.35	0.101	-0.34	0.1218	-0.4	0.1028
1-palmitoyl-GPC (16:0)	-0.42	0.0373	-0.34	0.1094	-0.34	0.1251	-0.4	0.104
glycoursodeoxycholate	0.25	0.0381	0.27	0.0311	0.26	0.0424	0.24	0.1932
urea	0.63	0.039	0.49	0.1241	0.44	0.1626	1.08	0.0146
N6-carbamoylthreonyladenosine	0.57	0.0392	0.43	0.157	0.45	0.1569	0.49	0.1557
1-palmitoleoyl-GPC (16:1)	-0.35	0.0409	-0.29	0.1004	-0.32	0.0915	-0.23	0.3239
glycerate	0.89	0.0432	0.77	0.0857	0.83	0.0765	2	0.0132
erythritol	0.6	0.0435	0.45	0.1657	0.46	0.1633	0.66	0.1146
1-stearoyl-GPC (18:0)	-0.36	0.0443	-0.3	0.1108	-0.29	0.1395	-0.33	0.1251
arabitol	0.49	0.0501	0.37	0.158	0.37	0.1676	0.54	0.1306
xylonate	0.35	0.0517	0.26	0.18	0.29	0.1466	0.25	0.3219
2-hydroxyglutarate	0.58	0.0531	0.57	0.0662	0.59	0.0625	1.02	0.0435
creatine	0.44	0.0533	0.39	0.0998	0.33	0.1796	0.51	0.1233
docosapentaenoate (n6 DPA; 22:5n6)	-0.59	0.0545	-0.51	0.1085	-0.41	0.2091	-0.68	0.1015
docosapentaenoate (DPA; 22:5n3)	-0.66	0.0555	-0.62	0.0852	-0.59	0.1161	-0.93	0.0591
linolenate (18:3n3 or 3n6)	-0.53	0.0576	-0.57	0.0531	-0.65	0.039	-0.58	0.1559
cysteine	0.52	0.0579	0.45	0.1012	0.41	0.1422	1.01	0.0358
N-acetylaspartate (NAA)	0.43	0.0607	0.45	0.0584	0.4	0.1017	0.65	0.0692
hexadecanedioate (C16)	0.42	0.0699	0.47	0.0485	0.43	0.0748	0.44	0.1762
N-acetylneuraminate	0.49	0.0797	0.33	0.2922	0.41	0.2049	0.3	0.3939
3-hydroxy-2-ethylpropionate	0.52	0.0812	0.51	0.0982	0.54	0.0864	0.47	0.3381
pseudouridine	0.61	0.0839	0.43	0.2559	0.43	0.2543	0.59	0.1908
4-acetamidobutanoate	0.46	0.0863	0.31	0.291	0.34	0.2578	0.45	0.2005
malate	0.45	0.0898	0.47	0.09	0.45	0.1056	0.8	0.0637
gamma-glutamylvaline	0.66	0.0919	0.52	0.194	0.59	0.168	1.17	0.0573
gluconate	0.15	0.0952	0.09	0.4304	0.08	0.4627	0.1	0.4644
2-hydroxybutyrate (AHB)	0.5	0.1147	0.54	0.0936	0.45	0.1813	0.81	0.1432
pipecolate	0.37	0.1185	0.36	0.1394	0.36	0.1475	0.52	0.1637
xylose	0.45	0.12	0.35	0.2433	0.5	0.1137	0.63	0.1089
bilirubin (E,E)	0.42	0.1226	0.65	0.0347	0.68	0.0362	0.53	0.1539
cystine	0.33	0.1238	0.33	0.1228	0.28	0.2086	0.24	0.4967
1-eicosatrienoyl-GPC (20:3)	-0.28	0.1253	-0.2	0.3015	-0.19	0.3414	-0.27	0.2181
1-arachidonoyl-GPI (20:4)	-0.54	0.1265	-0.61	0.0949	-0.69	0.0756	-0.32	0.5292
tryptophan	0.62	0.1357	0.78	0.092	0.76	0.0949	1.57	0.0497
pyroglutamine	0.39	0.144	0.46	0.101	0.46	0.1161	0.48	0.2308

beta-sitosterol	0.28	0.1477	0.18	0.3805	0.15	0.4736	0.47	0.0635
3-dehydrocarnitine	0.47	0.1504	0.45	0.1868	0.5	0.1635	1.24	0.0337
glutamate	-0.35	0.1543	-0.33	0.1821	-0.29	0.2497	-0.58	0.1074
threitol	0.33	0.1641	0.21	0.3864	0.26	0.3041	0.37	0.2228
arabinose	0.48	0.1644	0.33	0.3594	0.36	0.3385	0.86	0.0965
hippurate	0.21	0.1703	0.11	0.5307	0.11	0.537	0.18	0.4113
C-glycosyltryptophan	0.45	0.1812	0.24	0.5042	0.26	0.4832	0.34	0.4118
octanoylcarnitine (C8)	0.45	0.1967	0.41	0.2393	0.33	0.3665	0.85	0.0871
vanillylmandelate (VMA)	0.29	0.2028	0.15	0.5493	0.18	0.5026	0.4	0.2019
phenol sulfate	0.19	0.2085	0.12	0.442	0.26	0.1348	0.22	0.2089
acetoacetate	-0.22	0.209	-0.23	0.1915	-0.21	0.2463	-0.27	0.2461
3-carboxy-4-methyl-5-propyl-2-furanpropanoate (CMPF)	-0.18	0.2114	-0.18	0.2204	-0.15	0.3408	-0.16	0.3602
octadecanedioate (C18)	0.27	0.2167	0.33	0.1477	0.29	0.1964	0.22	0.4818
hypoxanthine	0.31	0.2175	0.33	0.2091	0.35	0.2097	0.75	0.0592
4-androsten-3beta,17beta-diol disulfate 2	0.26	0.219	0.17	0.4389	0.2	0.3834	0.46	0.1312
mannitol	0.14	0.2193	0.07	0.6106	0.09	0.488	0.11	0.4169
pantothenate (Vitamin B5)	0.39	0.223	0.25	0.4573	0.43	0.2451	0.24	0.5902
urate	0.56	0.2269	0.56	0.2119	0.75	0.1327	0.86	0.1786
N-acetylthreonine	0.4	0.2316	0.23	0.5051	0.21	0.5447	0.42	0.343
lathosterol	-0.37	0.2333	-0.47	0.1612	-0.38	0.2512	-0.39	0.3972
serine	-0.38	0.2376	-0.32	0.332	-0.28	0.4017	-0.52	0.276
pregnen-diol disulfate	0.21	0.2378	0.13	0.475	0.12	0.5379	0.4	0.1275
glutaroylcarnitine (C5)	0.35	0.2424	0.13	0.7024	0.3	0.4025	0.73	0.0734
deoxycarnitine	0.42	0.2427	0.4	0.2831	0.37	0.3261	0.91	0.0999
threonine	0.37	0.2463	0.43	0.1949	0.52	0.1546	0.33	0.4992
caffeine	0.17	0.256	0.28	0.0752	0.27	0.0967	0.26	0.1268
pyridoxate	0.17	0.2602	0.06	0.7166	0.13	0.4599	0.2	0.3982
heptanoate (7:0)	-0.4	0.2605	-0.31	0.3978	-0.24	0.5162	-0.78	0.1896
fructose	-0.24	0.283	-0.22	0.3461	-0.11	0.6477	0.11	0.6914
glycerol	-0.39	0.2842	-0.54	0.159	-0.57	0.1595	0.17	0.7381
pelargonate (9:0)	-0.37	0.2862	-0.27	0.4297	-0.24	0.489	-0.56	0.2895
hydroxyproline	0.34	0.2865	0.51	0.1283	0.52	0.1369	-0.08	0.8782
5-dodecenoate (12:1n7)	0.34	0.2941	0.5	0.1569	0.4	0.2842	0.57	0.1828
stachydrine	-0.13	0.2949	-0.12	0.3524	-0.1	0.4757	-0.2	0.2141
4-vinylphenol sulfate	-0.16	0.3037	-0.17	0.3122	-0.14	0.4401	-0.48	0.0908
pregn steroid monosulfate	0.18	0.314	0.16	0.4008	0.18	0.3595	0.36	0.2089
docosahexaenoate (DHA; 22:6n3)	-0.34	0.3152	-0.23	0.5278	-0.15	0.6873	-0.34	0.4442
2-methoxyacetaminophen sulfate	0.1	0.3326	0.1	0.339	0.08	0.4422	0.08	0.5078
phenylacetate	-0.25	0.3385	-0.31	0.2471	-0.23	0.4249	-0.13	0.7475
iminodiacetate (IDA)	-0.23	0.3526	-0.29	0.2671	-0.24	0.3576	0.25	0.5481
dihomolinoleate (20:2n6)	-0.3	0.3661	-0.26	0.4747	-0.22	0.561	-0.23	0.6219
1-stearoylglycerol (18:0)	0.31	0.3771	0.28	0.4324	0.28	0.4611	0.38	0.4542
proline	0.31	0.4018	0.33	0.3728	0.41	0.3004	0.66	0.4072
phenylacetylglutamine	0.14	0.4104	0.07	0.6676	0.18	0.3598	0.13	0.5923

3-(cystein-S-yl)acetaminophen	0.07	0.4352	0.07	0.4682	0.04	0.6938	0.05	0.672
1,6-anhydroglucose	0.13	0.4392	0.11	0.5352	0.11	0.5327	0.19	0.3801
3-methylhistidine	-0.13	0.4434	-0.12	0.4763	-0.09	0.6473	-0.28	0.2596
arginine	-0.17	0.4487	-0.13	0.5533	-0.12	0.6017	0.02	0.9569
epiandrosterone sulfate	-0.12	0.4535	-0.18	0.2719	-0.13	0.474	-0.02	0.9303
5alpha-pregnan-3beta,20alpha-diol disulfate	0.1	0.4545	0.04	0.8	0.08	0.6095	0.21	0.2639
3-indoxyl sulfate	-0.1	0.4608	-0.12	0.3609	-0.07	0.649	-0.07	0.695
5alpha-androstan-3beta,17beta-diol disulfate	0.15	0.4835	0.06	0.7976	0.07	0.744	0.34	0.1917
caprylate (8:0)	0.24	0.4844	0.31	0.3619	0.44	0.2393	0.13	0.7555
quinate	0.11	0.4903	0.12	0.4481	0.16	0.3676	-0.01	0.9725
choline	0.34	0.4956	0.14	0.791	0.12	0.8293	0.22	0.7618
scyllo-inositol	0.12	0.5034	0.05	0.7664	0.07	0.7057	0.03	0.9018
N-acetylglycine	-0.21	0.5056	-0.2	0.5338	-0.2	0.5485	-0.17	0.6907
3-methyl-2-oxovalerate	-0.26	0.5208	0.04	0.9358	0	0.9941	-0.28	0.5961
vaccenate (18:1n7)	-0.25	0.5246	-0.17	0.6731	-0.12	0.774	-0.28	0.6109
alpha-ketoglutarate	-0.18	0.5273	-0.22	0.4524	-0.24	0.4466	0.21	0.6404
piperine	-0.14	0.5358	-0.09	0.6794	-0.04	0.8659	-0.08	0.7723
palmitoleate (16:1n7)	-0.16	0.5574	-0.07	0.831	-0.13	0.6814	-0.21	0.5684
caprate (10:0)	0.22	0.5699	0.35	0.362	0.43	0.2979	0.03	0.9388
uridine	-0.22	0.5792	-0.04	0.9223	-0.08	0.8486	-0.18	0.7288
succinylcarnitine	0.19	0.5913	0.01	0.989	0.13	0.7482	0.22	0.6044
pyruvate	-0.18	0.6134	-0.14	0.7061	-0.03	0.9355	-0.12	0.7956
AMP	-0.17	0.6236	-0.14	0.6824	-0.09	0.806	-0.63	0.2368
citrate	0.18	0.6247	0.35	0.3742	0.46	0.2803	0.46	0.4089
4-acetamidophenol	0.05	0.6287	0.05	0.6148	0.02	0.8487	0.04	0.7772
betaine	0.14	0.633	0.2	0.5115	0.2	0.5227	-0.27	0.4941
creatinine	0.19	0.6486	-0.11	0.8116	0.14	0.7839	0.25	0.6439
4-androsten-3beta,17beta-diol disulfate 1	0.08	0.6517	0.01	0.9435	-0.03	0.878	0.08	0.7341
10-heptadecenoate (17:1n7)	-0.13	0.654	-0.02	0.9561	-0.03	0.9188	-0.28	0.4867
cis-4-decenoyl carnitine	0.14	0.6756	0.01	0.975	-0.07	0.8446	0.7	0.1459
alpha-ketobutyrate	-0.13	0.6764	-0.05	0.8647	-0.06	0.8614	-0.12	0.7793
3-hydroxybutyrate (BHBA)	-0.07	0.6815	-0.09	0.6026	-0.08	0.6393	-0.2	0.3508
palmitoylcarnitine (C16)	-0.09	0.6818	0.01	0.9614	0.02	0.9187	-0.09	0.7528
glycerol 3-phosphate (G3P)	-0.14	0.6844	-0.15	0.674	-0.13	0.7187	-0.49	0.3294
laurylcarnitine (C12)	0.09	0.6853	0.15	0.4998	0.12	0.6109	0.32	0.3046
2-hydroxyacetaminophen sulfate	0.04	0.748	0.03	0.8405	0.01	0.9107	0.04	0.7934
2-aminobutyrate	-0.1	0.7587	-0.02	0.9507	-0.15	0.6898	0.23	0.7075
p-acetamidophenylglucuronide	-0.03	0.7608	-0.04	0.7255	-0.05	0.6412	-0.03	0.8225
myo-inositol	0.06	0.7678	-0.02	0.9305	0	0.9829	0.01	0.959
eicosenoate (20:1n9 or 1n11)	-0.09	0.7729	-0.05	0.8911	0	0.9962	-0.29	0.528
margarate (17:0)	-0.11	0.774	0.01	0.972	0.02	0.9584	-0.55	0.3427
dehydroisoandrosterone sulfate (DHEA-S)	-0.04	0.7934	-0.1	0.5558	-0.11	0.5543	0.04	0.8568
prolylhydroxyproline	-0.07	0.7936	-0.17	0.5294	-0.14	0.619	-0.04	0.9009
androsterone sulfate	-0.04	0.8021	-0.11	0.4731	-0.09	0.5536	0.04	0.8502

isovalerate (C5)	-0.1	0.8031	0.07	0.8686	0.12	0.7904	0.35	0.5131
maltose	0.06	0.8062	0.01	0.983	0.09	0.7467	0.27	0.5068
4-acetaminophen sulfate	0.02	0.82	0.01	0.8772	0.01	0.9249	0	0.9636
catechol sulfate	-0.03	0.8387	0.04	0.8191	0.14	0.4817	-0.31	0.1733
citrulline	0.06	0.8406	0.08	0.8044	0.17	0.6274	0.16	0.7446
myristoleate (14:1n5)	-0.05	0.853	0.06	0.8325	-0.02	0.943	0.03	0.9347
10-nonadecenoate (19:1n9)	-0.05	0.853	0.03	0.922	0.04	0.8957	-0.13	0.754
heme	0.03	0.8603	-0.03	0.8885	-0.05	0.815	0.17	0.519
alanine	0.06	0.865	0.13	0.7143	0.16	0.6605	0.12	0.7993
1,5-anhydroglucitol (1,5-AG)	0.03	0.8769	0.13	0.5509	0.08	0.752	-0.22	0.4384
myristate (14:0)	-0.07	0.8784	0.16	0.7456	0.04	0.9421	-0.4	0.4909
4-methyl-2-oxopentanoate	-0.05	0.8933	0.2	0.6011	0.28	0.4881	0	0.9955
p-cresol sulfate	0.01	0.9278	0	0.9813	0.06	0.5302	-0.08	0.4961
threonate	-0.02	0.9486	-0.14	0.6219	-0.02	0.9473	0.08	0.8185
deoxycholate	0.01	0.9554	0.06	0.6885	0.11	0.4976	0.16	0.4034
decanoylcarnitine (C10)	0.01	0.9699	0.07	0.8456	0.02	0.9694	0.28	0.5473
