# Supplementary material for: Metabolomic Derangements Are Associated with Mortality in Critically Ill Adult Patients
Source: PLoS One. 2014 Jan 30;9(1):e87538. doi: 10.1371/journal.pone.0087538 (PMC3907548; doi:10.1371/journal.pone.0087538)
Supplement: Table S2 — 31 metabolites associated with mortality in both cohorts. 1Metabolite class is defined by Metabolon, inc. 2P value and β are for association of a given metabolite with 28-day mortality in the RoCI cohort, using logistic regression after adjustment for age, gender, race, malignancy status, and renal function. Metabolite values are log2-transformed for testing. 3P value in CAPSOD is similarly for logistic regression after the same adjustments. P values are 1-sided, as only metabolites with consistent direction are considered replicated. (PDF) [file pone.0087538.s002.pdf]

Supplemental Table S2. 31 metabolites associated with mortality in both cohorts

Metabolite	Class[1]	RoCI β[2]	RoCI P value[2]	CAPSOD β[3]	CAPSOD P value[3]
1-arachidonoyl-GPE (20:4)	Lipid	-1.51	1.00E-04	-0.41	0.0142
3-(4-hydroxyphenyl)lactate (HPLA)	Amino acid	1.09	3.00E-04	0.86	1.3E-05
taurochenodeoxycholate	Lipid	0.59	7.00E-04	0.22	0.0028
taurocholate	Lipid	0.48	0.0015	0.21	0.0077
gamma-glutamylphenylalanine	Peptide	1.59	0.0017	0.61	0.0272
glycochenodeoxycholate	Lipid	0.58	0.002	0.19	0.0114
1-arachidonoyl-GPC (20:4)	Lipid	-0.56	0.0032	-0.3	0.0016
glycocholate	Lipid	0.53	0.0036	0.18	0.0259
hydroxyisovaleroylcarnitine (C5)	Amino acid	0.79	0.0041	0.4	0.0317
hexanoylcarnitine (C6)	Lipid	1.15	0.0053	0.97	6.6E-05
lactate	Carbohydrate	1.11	0.0071	0.69	0.0084
alpha-hydroxyisovalerate	Amino acid	0.72	0.0071	0.58	3.8E-04
1-methylimidazoleacetate	Amino acid	0.63	0.0082	0.38	0.0104
isobutyrylcarnitine (C4)	Amino acid	0.71	0.0095	0.34	0.034
beta-hydroxyisovalerate	Amino acid	0.83	0.0134	0.37	0.0364
kynurenate	Amino acid	0.46	0.0137	0.37	0.0494
2-methylbutyroylcarnitine (C5)	Amino acid	0.75	0.0142	0.59	0.0024
1-linoleoyl-GPC (18:2)	Lipid	-0.42	0.0155	-0.3	0.0162
propionylcarnitine (C3)	Lipid	0.9	0.0156	0.62	0.0013
cortisol	Lipid	0.62	0.0175	0.56	0.0118
allantoin	Nucleotide	0.52	0.0191	0.55	0.0124
N2,N2-dimethylguanosine	Nucleotide	0.67	0.0213	0.5	0.0071
tiglyl carnitine (C5)	Amino acid	0.7	0.0219	0.72	0.0054
xanthine	Nucleotide	0.51	0.022	0.55	0.0204
N-acetylalanine	Amino acid	1.32	0.0243	1.03	0.004
N-acetylserine	Amino acid	0.71	0.0263	0.65	0.0028
butyrylcarnitine (C4)	Lipid	0.81	0.0306	0.75	3.6E-04
ornithine	Amino acid	0.51	0.0361	0.3	0.0232
2-palmitoyl-GPC (16:0)	Lipid	-0.42	0.0365	-0.31	0.0482
1-palmitoyl-GPC (16:0)	Lipid	-0.42	0.0373	-0.42	0.002
1-stearoyl-GPC (18:0)	Lipid	-0.36	0.0443	-0.26	0.0324
