# Supplementary material for: Metabolomic Derangements Are Associated with Mortality in Critically Ill Adult Patients
Source: PLoS One. 2014 Jan 30;9(1):e87538. doi: 10.1371/journal.pone.0087538 (PMC3907548; doi:10.1371/journal.pone.0087538)
Supplement: Table S3 — Association with mortality in all 308 Metabolites tested in RoCI. Y: Metabolite tested only in RoCI, not in CAPSOD. P values and β are for association of a given metabolite with 28-day mortality in the RoCI cohort, using logistic regression after adjustment for age, gender, race, malignancy status, and renal function. Metabolite values are log2-transformed for testing. (PDF) [file pone.0087538.s003.pdf]

Supplemental Table S3. Association with mortality in all 308 Metabolites tested in RoCI

Metabolite	Class	RoCI β[1]	P value[1]	Tested only in RoCI
1-arachidonoyl-GPE (20:4)	Lipid	-1.51	1.00E-04	
3-(4-hydroxyphenyl)lactate (HPLA)	Amino acid	1.09	3.00E-04	
gamma-glutamyltyrosine	Peptide	2.06	5.00E-04	
phenyllactate (PLA)	Amino acid	1.15	6.00E-04	Y
taurochenodeoxycholate	Lipid	0.59	7.00E-04	
kynurenine	Amino acid	1.54	0.0012	
3-hydroxyisobutyrate	Amino acid	1.89	0.0012	Y
sucrose	Carbohydrate	0.35	0.0014	
taurocholate	Lipid	0.48	0.0015	
gamma-glutamylphenylalanine	Peptide	1.59	0.0017	
glycochenodeoxycholate	Lipid	0.58	0.002	
N-acetyltyrosine	Amino acid	0.85	0.0021	Y
ribitol	Carbohydrate	1.84	0.0024	Y
indolelactate	Amino acid	0.95	0.0024	
1-oleoyl-GPE (18:1)	Lipid	-1.05	0.0032	
1-arachidonoyl-GPC (20:4)	Lipid	-0.56	0.0032	
1-linoleoyl-GPE (18:2)	Lipid	-1	0.0032	Y
glycocholenate sulfate	Lipid	0.63	0.0033	
glycocholate	Lipid	0.53	0.0036	
mannose	Carbohydrate	-1.36	0.004	
hydroxyisovaleroylcarnitine (C5)	Amino acid	0.79	0.0041	
stearidonate (18:4n3)	Lipid	-0.88	0.0043	
trans-urocanate	Amino acid	0.49	0.0048	Y
hexanoylcarnitine (C6)	Lipid	1.15	0.0053	
fucose	Carbohydrate	0.9	0.0056	Y
2-hydroxy-3-methylvalerate	Amino acid	0.74	0.0059	Y
isovalerylcarnitine (C5)	Amino acid	0.82	0.006	
N-6-trimethyllysine	Amino acid	1.03	0.0061	Y
lactate	Carbohydrate	1.11	0.0071	
alpha-hydroxyisovalerate	Amino acid	0.72	0.0071	
3-ureidopropionate	Amino acid	0.84	0.0077	Y
cortisone	Lipid	0.94	0.0079	Y
7-methylguanine	Nucleotide	1.26	0.0079	Y
1-methylimidazoleacetate	Amino acid	0.63	0.0082	
2-arachidonoyl-GPE (20:4)	Lipid	-0.9	0.0091	Y
gamma-glutamylmethionine	Peptide	0.75	0.0095	Y
glycolithocholate sulfate	Lipid	0.39	0.0095	
isobutyrylcarnitine (C4)	Amino acid	0.71	0.0095	
taurocholenate sulfate	Lipid	0.42	0.0101	
13-HODE + 9-HODE	Lipid	-0.71	0.0124	Y

biliverdin	Cofactors and vitamins	0.72	0.013	
beta-hydroxyisovalerate	Amino acid	0.83	0.0134	
1-docosahexaenoyl-GPC (22:6)	Lipid	-0.48	0.0134	Y
kynurenate	Amino acid	0.46	0.0137	
2-methylbutyroylcarnitine (C5)	Amino acid	0.75	0.0142	
1-palmitoyl-GPE (16:0)	Lipid	-0.91	0.0142	Y
dihomolinolenate (20:3n3 or 3n6)	Lipid	-1.23	0.0151	
1-linoleoyl-GPC (18:2)	Lipid	-0.42	0.0155	
propionylcarnitine (C3)	Lipid	0.9	0.0156	
ibuprofen	Xenobiotics	0.45	0.0157	Y
pregnenolone sulfate	Lipid	0.51	0.0172	Y
cortisol	Lipid	0.62	0.0175	
indoleacetate	Amino acid	0.82	0.0178	Y
allantoin	Nucleotide	0.52	0.0191	
N-acetylphenylalanine	Amino acid	0.7	0.0196	Y
N2,N2-dimethylguanosine	Nucleotide	0.67	0.0213	
1-oleoyl-GPC (18:1)	Lipid	-0.43	0.0216	
glucarate (saccharate)	Cofactors and vitamins	0.25	0.0216	Y
tiglyl carnitine (C5)	Amino acid	0.7	0.0219	
xanthine	Nucleotide	0.51	0.022	
N-acetylalanine	Amino acid	1.32	0.0243	
tauroursodeoxycholate	Lipid	0.56	0.025	Y
N-acetylserine	Amino acid	0.71	0.0263	
taurolithocholate 3-sulfate	Lipid	0.33	0.027	
1-stearoyl-GPE (18:0)	Lipid	-0.67	0.0275	Y
gamma-tocopherol	Cofactors and vitamins	-0.5	0.0287	Y
methionine	Amino acid	0.96	0.029	
2-myristoyl-GPC (14:0)	Lipid	-0.4	0.0292	Y
estrone 3-sulfate	Lipid	0.4	0.0301	Y
erythronate	Carbohydrate	0.59	0.0306	
butyrylcarnitine (C4)	Lipid	0.81	0.0306	
5-methylthioadenosine (MTA)	Amino acid	0.74	0.0307	Y
glucose	Carbohydrate	-1.23	0.0315	
sphingomyelin	Lipid	-0.75	0.0331	Y
glucuronate	Carbohydrate	0.36	0.0333	
1-docosapentaenoyl-GPC (22:5n3)	Lipid	-0.63	0.0337	Y
bilirubin	Cofactors and vitamins	0.38	0.0348	
ofloxacin	Xenobiotics	0.13	0.0352	Y
ornithine	Amino acid	0.51	0.0361	
2-palmitoyl-GPC (16:0)	Lipid	-0.42	0.0365	

1-palmitoyl-GPC (16:0)	Lipid	-0.42	0.0373	
1-palmitoyl-GPI (16:0)	Lipid	0.65	0.0378	Y
glycoursodeoxycholate	Lipid	0.25	0.0381	
urea	Amino acid	0.63	0.039	
N6-carbamoylthreonyladenosine	Nucleotide	0.57	0.0392	
1-palmitoleoyl-GPC (16:1)	Lipid	-0.35	0.0409	
glycerate	Carbohydrate	0.89	0.0432	
erythritol	Xenobiotics	0.6	0.0435	
1-stearoyl-GPC (18:0)	Lipid	-0.36	0.0443	
1,2-propanediol	Lipid	0.21	0.0474	Y
1-oleoylglycerol (18:1)	Lipid	-0.42	0.0475	Y
fumarate	Energy	0.91	0.0488	Y
1-palmitoylplasmenylethanolamine	Lipid	-0.58	0.0498	Y
arabitol	Carbohydrate	0.49	0.0501	
xylonate	Carbohydrate	0.35	0.0517	
theobromine	Xenobiotics	0.35	0.0528	Y
2-hydroxyglutarate	Lipid	0.58	0.0531	
creatine	Amino acid	0.44	0.0533	
sarcosine (N-Methylglycine)	Amino acid	0.45	0.0543	Y
docosapentaenoate (n6 DPA; 22:5n6)	Lipid	-0.59	0.0545	
ascorbate (Vitamin C)	Cofactors and vitamins	-0.36	0.0554	Y
docosapentaenoate (DPA; 22:5n3)	Lipid	-0.66	0.0555	
linolenate (18:3n3 or 3n6)	Lipid	-0.53	0.0576	
cysteine	Amino acid	0.52	0.0579	
N-acetylaspartate (NAA)	Amino acid	0.43	0.0607	
serotonin (5HT)	Amino acid	-0.58	0.0608	Y
maltotriose	Carbohydrate	-0.58	0.0611	Y
gamma-glutamylisoleucine	Peptide	0.78	0.062	Y
2-hydroxyisobutyrate	Amino acid	0.4	0.0644	Y
hexadecanedioate (C16)	Lipid	0.42	0.0699	
xylitol	Carbohydrate	0.43	0.0748	Y
N-acetylneuraminate	Carbohydrate	0.49	0.0797	
3-hydroxy-2-ethylpropionate	Amino acid	0.52	0.0812	
pseudouridine	Nucleotide	0.61	0.0839	
2-linoleoyl-GPE (18:2)	Lipid	-0.69	0.084	Y
4-acetamidobutanoate	Amino acid	0.46	0.0863	
arabonate	Cofactors and vitamins	0.41	0.0878	Y
malate	Energy	0.45	0.0898	
gamma-glutamylvaline	Peptide	0.66	0.0919	
gluconate	Carbohydrate	0.15	0.0952	
tetradecanedioate (C14)	Lipid	0.4	0.0958	Y

tartarate	Xenobiotics	0.26	0.0977	Y
cortodoxone	Lipid	0.41	0.1047	Y
N4-acetylcytidine	Nucleotide	0.65	0.11	Y
2-hydroxybutyrate (AHB)	Amino acid	0.5	0.1147	
3-hydroxyoctanoate	Lipid	0.57	0.1156	Y
4-hydroxyphenylpyruvate	Amino acid	0.5	0.1176	Y
pipecolate	Amino acid	0.37	0.1185	
xylose	Carbohydrate	0.45	0.12	
bilirubin (E,E)	Cofactors and vitamins	0.42	0.1226	
cystine	Amino acid	0.33	0.1238	
1-eicosatrienoyl-GPC (20:3)	Lipid	-0.28	0.1253	
1-arachidonoyl-GPI (20:4)	Lipid	-0.54	0.1265	
21-hydroxypregnenolone disulfate	Lipid	0.3	0.1279	Y
N-acetyl-beta-alanine	Amino acid	0.61	0.1288	Y
5alpha-pregnan-3alpha,20beta-diol disulfate 1	Lipid	0.21	0.1295	Y
salicyluric glucuronide	Xenobiotics	-0.21	0.1313	Y
tryptophan	Amino acid	0.62	0.1357	
alpha-hydroxyisocaproate	Amino acid	0.37	0.1409	Y
pyroglutamine	Amino acid	0.39	0.144	
erythrulose	Carbohydrate	-0.32	0.1448	Y
beta-sitosterol	Lipid	0.28	0.1477	
3-dehydrocarnitine	Lipid	0.47	0.1504	
3-aminoisobutyrate	Nucleotide	0.26	0.1538	Y
glutamate	Amino acid	-0.35	0.1543	
1-heptadecanoyl-GPC (17:0)	Lipid	-0.28	0.1601	Y
N-methyl proline	Amino acid	-0.31	0.1617	Y
2-stearoyl-GPC (18:0)	Lipid	-0.26	0.1634	Y
threitol	Carbohydrate	0.33	0.1641	
arabinose	Carbohydrate	0.48	0.1644	
hippurate	Xenobiotics	0.21	0.1703	
valerate (5:0)	Lipid	-0.45	0.1753	Y
1-eicosadienoyl-GPC (20:2)	Lipid	-0.29	0.181	Y
C-glycosyltryptophan	Amino acid	0.45	0.1812	
N1-Methyl-2-pyridone-5-carboxamide	Cofactors and vitamins	0.29	0.1857	Y
campesterol	Lipid	0.48	0.1901	Y
octanoylcarnitine (C8)	Lipid	0.45	0.1967	
2-linoleoyl-GPC (18:2)	Lipid	-0.32	0.1989	Y
vanillylmandelate (VMA)	Amino acid	0.29	0.2028	
phenol sulfate	Amino acid	0.19	0.2085	
acetoacetate	Lipid	-0.22	0.209	
2-	Lipid	-0.31	0.209	Y

docosahexaenoylglycerophosphoethanolamine				
3-carboxy-4-methyl-5-propyl-2-furanpropanoate (CMPF)	Lipid	-0.18	0.2114	
5alpha-androstan-3alpha,17beta-diol disulfate	Lipid	0.21	0.2135	Y
octadecanedioate (C18)	Lipid	0.27	0.2167	
hypoxanthine	Nucleotide	0.31	0.2175	
beta-hydroxypyruvate	Amino acid	-0.42	0.2188	Y
4-androsten-3beta,17beta-diol disulfate 2	Lipid	0.26	0.219	
trizma acetate	Xenobiotics	-0.09	0.2192	Y
mannitol	Carbohydrate	0.14	0.2193	
4-hydroxyphenylacetate	Amino acid	0.21	0.2196	Y
tryptophan betaine	Amino acid	0.26	0.2228	Y
pantothenate (Vitamin B5)	Cofactors and vitamins	0.39	0.223	
urate	Nucleotide	0.56	0.2269	
N-acetylthreonine	Amino acid	0.4	0.2316	
2-hydroxyhippurate (salicylurate)	Xenobiotics	-0.19	0.2328	Y
lathosterol	Lipid	-0.37	0.2333	
serine	Amino acid	-0.38	0.2376	
pregnen-diol disulfate	Lipid	0.21	0.2378	
glutaroylcarnitine (C5)	Amino acid	0.35	0.2424	
deoxycarnitine	Lipid	0.42	0.2427	
cholate	Lipid	0.18	0.2456	Y
threonine	Amino acid	0.37	0.2463	
adipate	Lipid	0.25	0.2538	Y
caffeine	Xenobiotics	0.17	0.256	
pyridoxate	Cofactors and vitamins	0.17	0.2602	
heptanoate (7:0)	Lipid	-0.4	0.2605	
paraxanthine	Xenobiotics	0.18	0.2697	Y
fructose	Carbohydrate	-0.24	0.283	
glycerol	Lipid	-0.39	0.2842	
pelargonate (9:0)	Lipid	-0.37	0.2862	
hydroxyproline	Amino acid	0.34	0.2865	
3-hydroxydecanoate	Lipid	0.32	0.2889	Y
5-dodecenoate (12:1n7)	Lipid	0.34	0.2941	
stachydrine	Xenobiotics	-0.13	0.2949	
2-hydroxyphenylacetate	Xenobiotics	0.23	0.2954	Y
4-vinylphenol sulfate	Xenobiotics	-0.16	0.3037	
pregn steroid monosulfate	Lipid	0.18	0.314	
docosahexaenoate (DHA; 22:6n3)	Lipid	-0.34	0.3152	
cholestanol	Lipid	0.27	0.3217	Y

nicotinamide	Cofactors and vitamins	-0.34	0.3222	Y
2-methoxyacetaminophen sulfate	Xenobiotics	0.1	0.3326	
phenylacetate	Amino acid	-0.25	0.3385	
ursodeoxycholate	Lipid	0.14	0.3388	Y
N-formylmethionine	Amino acid	0.4	0.3456	Y
I-urobilinogen	Cofactors and vitamins	0.13	0.3463	Y
iminodiacetate (IDA)	Xenobiotics	-0.23	0.3526	
dihomolinoleate (20:2n6)	Lipid	-0.3	0.3661	
1-stearoylglycerol (18:0)	Lipid	0.31	0.3771	
beta-alanine	Amino acid	0.19	0.3983	Y
proline	Amino acid	0.31	0.4018	
phenylacetylglutamine	Amino acid	0.14	0.4104	
salicylate	Xenobiotics	-0.13	0.4148	Y
3-methylglutaroylcarnitine (C6)	Amino acid	0.15	0.4207	Y
2-oleoyl-GPC (18:1)	Lipid	-0.17	0.4276	Y
theophylline	Xenobiotics	0.15	0.4295	Y
3-hydroxyhippurate	Xenobiotics	0.2	0.4331	Y
3-(cystein-S-yl)acetaminophen	Xenobiotics	0.07	0.4352	
1,6-anhydroglucose	Carbohydrate	0.13	0.4392	
pyrophosphate (PPi)	Energy	0.3	0.4417	Y
3-methylhistidine	Amino acid	-0.13	0.4434	
arginine	Amino acid	-0.17	0.4487	
sorbitol	Carbohydrate	0.14	0.4521	Y
hydantoin-5-propionic acid	Amino acid	0.2	0.4528	Y
epiandrosterone sulfate	Lipid	-0.12	0.4535	
5alpha-pregnan-3beta,20alpha-diol disulfate	Lipid	0.1	0.4545	
trigonelline (N'-methylnicotinate)	Cofactors and vitamins	-0.15	0.4592	Y
3-indoxyl sulfate	Amino acid	-0.1	0.4608	
thymol sulfate	Xenobiotics	0.22	0.4612	Y
5alpha-androstan-3beta,17beta-diol disulfate	Lipid	0.15	0.4835	
caprylate (8:0)	Lipid	0.24	0.4844	
quinate	Xenobiotics	0.11	0.4903	
choline	Lipid	0.34	0.4956	
scyllo-inositol	Lipid	0.12	0.5034	
N-acetylglycine	Amino acid	-0.21	0.5056	
3-methyl-2-oxovalerate	Amino acid	-0.26	0.5208	
vaccenate (18:1n7)	Lipid	-0.25	0.5246	
alpha-ketoglutarate	Energy	-0.18	0.5273	
glycerol 2-phosphate	Xenobiotics	-0.19	0.5355	Y

piperine	Xenobiotics	-0.14	0.5358	
chiro-inositol	Lipid	-0.1	0.5568	Y
palmitoleate (16:1n7)	Lipid	-0.16	0.5574	
N-acetylmethionine	Amino acid	-0.15	0.5618	Y
caprate (10:0)	Lipid	0.22	0.5699	
stearoylcarnitine (C18)	Lipid	-0.15	0.571	Y
uridine	Nucleotide	-0.22	0.5792	
succinylcarnitine	Energy	0.19	0.5913	
metoprolol acid metabolite	Xenobiotics	0.04	0.6091	Y
pyruvate	Carbohydrate	-0.18	0.6134	
cys-gly, oxidized	Peptide	-0.1	0.6194	Y
AMP	Nucleotide	-0.17	0.6236	
citrate	Energy	0.18	0.6247	
4-acetamidophenol	Xenobiotics	0.05	0.6287	
betaine	Amino acid	0.14	0.633	
glycerophosphorylcholine (GPC)	Lipid	-0.14	0.642	Y
creatinine	Amino acid	0.19	0.6486	
4-androsten-3beta,17beta-diol disulfate 1	Lipid	0.08	0.6517	
10-heptadecenoate (17:1n7)	Lipid	-0.13	0.654	
gamma-CEHC	Cofactors and vitamins	-0.13	0.6692	Y
cis-4-decenoyl carnitine	Lipid	0.14	0.6756	
alpha-ketobutyrate	Amino acid	-0.13	0.6764	
3-hydroxybutyrate (BHBA)	Lipid	-0.07	0.6815	
palmitoylcarnitine (C16)	Lipid	-0.09	0.6818	
glycerol 3-phosphate (G3P)	Lipid	-0.14	0.6844	
laurylcarnitine (C12)	Lipid	0.09	0.6853	
4-hydroxyhippurate	Xenobiotics	0.08	0.696	Y
1-pentadecanoylglycerophosphocholine (15:0)	Lipid	-0.09	0.7227	Y
docosadienoate (22:2n6)	Lipid	0.11	0.7447	Y
3-phosphoglycerate	Carbohydrate	0.07	0.7465	Y
2-hydroxyacetaminophen sulfate	Xenobiotics	0.04	0.748	
2-aminobutyrate	Amino acid	-0.1	0.7587	
p-acetamidophenylglucuronide	Xenobiotics	-0.03	0.7608	
myo-inositol	Lipid	0.06	0.7678	
eicosenoate (20:1n9 or 1n11)	Lipid	-0.09	0.7729	
margarate (17:0)	Lipid	-0.11	0.774	
17-methylstearate	Lipid	-0.1	0.7885	Y
dehydroisoandrosterone sulfate (DHEA-S)	Lipid	-0.04	0.7934	
prolylhydroxyproline	Peptide	-0.07	0.7936	

saccharin	Xenobiotics	-0.04	0.7954	Y
androsterone sulfate	Lipid	-0.04	0.8021	
isovalerate (C5)	Lipid	-0.1	0.8031	
maltose	Carbohydrate	0.06	0.8062	
4-acetaminophen sulfate	Xenobiotics	0.02	0.82	
catechol sulfate	Xenobiotics	-0.03	0.8387	
citrulline	Amino acid	0.06	0.8406	
myristoleate (14:1n5)	Lipid	-0.05	0.853	
10-nonadecenoate (19:1n9)	Lipid	-0.05	0.853	
andro steroid monosulfate 2	Lipid	0.03	0.8537	Y
heme	Cofactors and vitamins	0.03	0.8603	
alanine	Amino acid	0.06	0.865	
homostachydrine	Xenobiotics	-0.05	0.8714	Y
1,5-anhydroglucitol (1,5-AG)	Carbohydrate	0.03	0.8769	
myristate (14:0)	Lipid	-0.07	0.8784	
2-ethylhexanoic acid	Xenobiotics	-0.04	0.8882	Y
4-methyl-2-oxopentanoate	Amino acid	-0.05	0.8933	
ADSGEGDFXAEGGGVR	Peptide	0.02	0.8967	Y
L-urobilin	Cofactors and vitamins	0.01	0.8998	Y
10-undecenoate (11:1n1)	Lipid	0.04	0.9032	Y
dimethylglycine	Amino acid	-0.03	0.9167	Y
2-arachidonoyl-GPC (20:4)	Lipid	-0.02	0.921	Y
vancomycin	Xenobiotics	0.01	0.9214	Y
p-cresol sulfate	Amino acid	0.01	0.9278	
oleoylcarnitine (C18)	Lipid	0.02	0.9387	Y
16-hydroxypalmitate	Lipid	0.02	0.9465	Y
threonate	Cofactors and vitamins	-0.02	0.9486	
deoxycholate	Lipid	0.01	0.9554	
decanoylcarnitine (C10)	Lipid	0.01	0.9699	
beta-tocopherol	Cofactors and vitamins	0.01	0.9768	Y
