# Supplementary material for: Metabolomic Derangements Are Associated with Mortality in Critically Ill Adult Patients
Source: PLoS One. 2014 Jan 30;9(1):e87538. doi: 10.1371/journal.pone.0087538 (PMC3907548; doi:10.1371/journal.pone.0087538)
Supplement: Methods S1 — (PDF) [file pone.0087538.s004.pdf]

Supplemental Methods S1

Supplementary Data: Populations

The BWH RoCI is approved by the Partners IRB committee (2008-P-000495). The protocol for recruitment for RoCI has been published in detail elsewhere.[1] Briefly, adult patient (over age 18) who is admitted to the BWH Medical Intensive Care Unit is eligible for inclusion in the RoCI within 72 hours of presentation, unless certain exclusion criteria are met (unable to provide consent due to cognitive dysfunction or no appropriate health care proxy, prior refusal, admission purely for comfort care, Jehova's Witness status, or a baseline hemoglobin <8 g/dL or hemoglobin <9 g/dL with either admission for active bleeding or with acute ischemia). Plasma is obtained on Days 1, 3, and 7 of enrollment. Extensive phenotypic data (including age, gender, key comorbidities, and APACHE II score), laboratory and radiologic data are recorded for all subjects. Classification of Systemic Inflammatory Response (SIRS), Sepsis, and ARDS is determined by a consensus panel of ICU physicians using the current disease classification. Mortality data was recorded using the Partners Research Patient Data Registry (RPDR), and the Social Security Data Index.

Between September 2008 and May 2010, 225 subjects were enrolled in the RoCI. Among these 225 subjects, 90 subjects were selected for metabolic profiling: 29 with SIRS, 30 with Sepsis, and 31 with sepsis-ARDS. Cases were selected for profiling based in part on IL-18 levels as part of a separate analysis[1] (sepsis and SIRS patients with low IL-18 levels, ARDS with high IL-18 levels; 31 of the 34 ARDS cases at that time were used). Cases were not selected with regards to risk of death or any known metabolic feature.

<u>CAPSOD Population</u>: The protocol for enrollment in the Community Acquired Pneumonia and Sepsis Outcome Diagnostics (CAPSOD) study has likewise been previously published.[2-4] Briefly, 1152 patients with sepsis (≥2 SIRS criteria and presumed infection) were enrolled in emergency rooms associated with 3 US Hospitals (Henry Ford Hospital, Duke University Medical Center, and Durham Veterans Administration Medical Center). Blood samples and extensive phenotypic and laboratory data were recorded at enrollment. Survival/death was the primary outcome. The validation set of 150 patients (13% of the total CAPSOD cohort) had five groups that reflected conventional concepts of sepsis progression as a pyramid. The number of subjects was governed by power to test associations with survival/death. The five groups were: day 28 sepsis survivors with uncomplicated courses (n=27), sepsis survivors who developed severe sepsis or septic shock by day 3 (n=25 and n=38, respectively), sepsis deaths (by day 28; n=31), and non-infected patients who exhibited SIRS criteria (SIRS-positive, "ill" controls, presumed septic at enrollment but later determined to have non-infectious reasons for SIRS; n=29).

Supplementary Data: Formation of Bayesian Network associated with 28-day mortality for analysis of metabolomics data

A Bayesian network (BN) is a data structure that encodes conditional probability distributions among variables of interest by using a graph composed of nodes and directed edges[5]. In a BN, variables in the domain are modeled as random variables and represented by nodes, and edges between them represent a statistical dependence of the child node on the parent node. Each node is annotated with the marginal distribution of the variable conditioned on the values of its parents, and this information can be used to predict the most likely values of variables in the BN. A conditional Gaussian Bayesian network (CGBN) is a type of BN that allows mixing discrete and continuous variables in the same network [6].

To apply CGBN methods to the RoCI and CAPSOD datasets, we $\log_2$ transformed metabolite values and normalized separately in the RoCI and CAPSOD datasets. The only other continuous variable, age, was also normalized. Demographic variables that were eligible for inclusion in the Bayesian network were age, sex, APACHE II score, and renal function (using the glomerular filtration rate estimated using the GFR-MDRD equation(5)). Race and malignancy status were excluded based on the large disparity in racial and cancer compositions of the RoCI and CAPSOD cohorts. Apache II score, while significantly different between the cohorts, was included as a potential predictor because of its existing role as a predictor of ICU outcomes.

We constructed a pheno-centric CGBN [7] based on predicting the 28-day mortality This is a predictive network model that starts with the phenotype as the independent variable and then tests each other variable for statistical dependence. The network makes a link from the phenotype to the variable if the probability of association is more likely than the probability of independence, a test known as the Bayes Factor [8]. The pheno-centric network is then completed by testing if those variables are dependent variables of other metabolites. This forms the Markov neighborhood of the phenotype node and, according to Bayesian network semantics [5], are the only variables that influence the distribution of the phenotype. We then used the pheno-centric network to predict the value of 28-day ICU mortality from the data. Such a pheno-centric network has two main benefits. First, it can allow prediction in domains where building a full BN over all the variables is computationally prohibitive. Second, it does not rely on network structure constraints and as such does not needlessly exclude many potential network structures from consideration.

We used the CGBayesNets package[9] [7] in MATLAB for all Bayesian and related statistical computations. We used 5-fold cross-validation on RoCI to arrive at hyper parameters for the Bayesian likelihood calculations. We performed 2500 bootstrap realizations of the RoCI dataset, and learned a pheno-centric CGBN for each bootstrap realization. From the sample of 2500 networks,

we built a consensus network by starting with the phenotype node and then adding, in sequence, the most frequent edge occurring in the bootstrap networks, and measuring the performance of that network on the dataset in cross-validation. This provides a way of estimating the value of adding each node to the network, and roughly the point of diminishing returns. We used a network with a total of seven predictive nodes, shown in **Figure 2**, to define the final network model, as adding further nodes did not increase the predictive performance in cross-validation.

Assessing Performance of Bayesian Networks: Predictive performance of each network was assessed by the convex hull [10] of the Area Under the Receiver Operator Characteristic Curve (AUC).[11] Statistically significant differences between two AUCs over the same data points were assessed by first measuring the variance of this quantity, assuming the difference follows a normal distribution, and using a standard t-test.[11] The variance is computed according to the nonparametric method described by DeLong et al.[12] Network structure was determined by the RoCI dataset and was tested in CAPSOD without any parameter refitting, which provides the most stringent possible test of model replication.

For comparison with the predictive model identified by Langley et al. [13], we computed a Bayesian network using the 7 predictive variables identified by Langley et al. Using these 7 variables (age, hematocrit, lactate, 2-methylbutyroylcarnitine (C5), butyrylcarnitine (C4), and hexanoylcarnitine (C6), cis-4-decenoyl carnitine) we constructed the pheno-centric network that maximized the posterior likelihood of the CAPSOD data, using the same priors and parameters as used above. We then tested this network for predictive accuracy in both RoCI and CAPSOD. This provided the most apt comparison between the predictive variables identified by Langley et al. and identified through our analysis.

Supplementary Data: Sample Processing

Sample Handling, RoCI and CAPSOD cohorts

In the RoCI cohort, blood samples were drawn and transferred into EDTA coated blood collection tubes within 24 hours from study inclusion and processed within 4 hours after venipuncture. Subsequently, plasma was fractionated and stored at -80°C as previously described [1]. 150 µl aliquots were shipped on dry-ice to Metabolon, Inc., without any accompanying clinical information. Following receipt, the frozen samples in the box were immediately stored at -80°C.

In CAPSOD study, blood for metabolomic and proteomic analyses was collected in bar-coded EDTA-plasma tubes at enrollment (t_0) and the following day (t_{24}), incubated on ice, plasma separated (within 4 hours), and aliquots stored at -80°C. At the time of analysis samples were extracted and prepared for analysis using Metabolon's standard solvent extraction method. [14] The extracted samples were split into equal parts for analysis on the GC/MS and LC/MS/MS platforms. Also included were several technical replicate samples created from a homogeneous pool containing a small amount of all study samples ("Client Matrix").

While metabolomic profiling for RoCI and CAPSOD was independently performed, identical methods for sample preparation were used in both studies.

Sample Accessioning: Each sample received was accessioned into the Metabolon LIMS system and was assigned by the LIMS a unique identifier, which was associated with the original source identifier only. This identifier was used to track all sample handling, tasks, results *etc.* The samples (and all derived aliquots) were bar-coded and tracked by the LIMS system. All portions of any sample were automatically assigned their own unique identifiers by the LIMS when a new task was created; the relationship of these samples was also tracked. All samples were maintained at -80 ºC until processed.

Sample Preparation: The sample preparation process was carried out using the automated MicroLab STAR® system from Hamilton Company. Recovery standards were added prior to the first step in the extraction process for QC purposes. Sample preparation was conducted using a proprietary series of organic and aqueous extractions to remove the protein fraction while allowing maximum recovery of small molecules. The resulting extract was divided into two fractions; one for analysis by LC and one for analysis by GC. Samples were placed briefly on a TurboVap® (Zymark) to remove the organic solvent. Each sample was then frozen and dried under vacuum. Samples were then prepared for the appropriate instrument, either LC/MS or GC/MS.

QA/QC: For QA/QC purposes, a number of additional samples are included with each day's analysis. Furthermore, a selection of QC compounds is added to every sample, including those under test. These compounds are carefully

chosen so as not to interfere with the measurement of the endogenous compounds. Tables 1 and 2 describe the QC samples and compounds. These QC samples are primarily used to evaluate the process control for each study as well as aiding in the data curation

Table 1: Description of Metabolon QC Samples

Type	Description	Purpose
MTRX	Large pool of human plasma maintained by Metabolon that has been characterized extensively.	Assure that all aspects of Metabolon process are operating within specifications.
CMTRX	Pool created by taking a small aliquot from every customer sample.	Assess the effect of a non-plasma matrix on the Metabolon process and distinguish biological variability from process variability.
PRCS	Aliquot of ultra-pure water	Process Blank used to assess the contribution to compound signals from the process.
SOLV	Aliquot of solvents used in extraction.	Solvent blank used to segregate contamination sources in the extraction.

Table 2: Metabolon QC Standards

Type	Description	Purpose
DS	Derivatization Standard	Assess variability of derivatization for GC/MS samples.
IS	Internal Standard	Assess variability and performance of instrument.
RS	Recovery Standard	Assess variability and verify performance of extraction and instrumentation.

Liquid chromatography/Mass Spectrometry (LC/MS, LC/MS^2): The LC/MS portion of the platform was based on a Waters ACQUITY UPLC and a Thermo-Finnigan LTQ mass spectrometer, which consisted of an electrospray ionization (ESI) source and linear ion-trap (LIT) mass analyzer. The sample extract was split into two aliquots, dried, then reconstituted in acidic or basic LC-compatible solvents, each of which contained 11 or more injection standards at fixed concentrations. One aliquot was analyzed using acidic positive ion optimized conditions and the other using basic negative ion optimized conditions in two independent injections using separate dedicated columns. Extracts reconstituted in acidic conditions were gradient eluted using water and methanol both containing 0.1% Formic acid, while the basic extracts, which also used water/methanol, contained 6.5mM Ammonium Bicarbonate. The MS analysis alternated between MS and data-dependent MS^2 scans using dynamic exclusion.

Gas chromatography/Mass Spectrometry (GC/MS): The samples destined for GC/MS analysis were re-dried under vacuum desiccation for a minimum of 24 hours prior to being derivatized under dried nitrogen using bistrimethyl-silyl-

triflouroacetamide (BSTFA). The GC column was 5% phenyl and the temperature ramp is from 40° to 300° C in a 16 minute period. Samples were analyzed on a Thermo-Finnigan Trace DSQ fast-scanning single-quadrupole mass spectrometer using electron impact ionization. The instrument was tuned and calibrated for mass resolution and mass accuracy on a daily basis. The information output from the raw data files was automatically extracted as discussed below.

Accurate Mass Determination and MS/MS fragmentation (LC/MS), (LC/MS/MS): The LC/MS portion of the platform was based on a Waters ACQUITY UPLC and a Thermo-Finnigan LTQ-FT mass spectrometer, which had a linear ion-trap (LIT) front end and a Fourier transform ion cyclotron resonance (FT-ICR) mass spectrometer backend. For ions with counts greater than 2 million, an accurate mass measurement could be performed. Accurate mass measurements could be made on the parent ion as well as fragments. The typical mass error was less than 5 ppm. Ions with less than two million counts require a greater amount of effort to characterize. Fragmentation spectra (MS/MS) were typically generated in data dependent manner, but if necessary, targeted MS/MS could be employed, such as in the case of lower level signals.

Bioinformatics: The informatics system consisted of four major components, the Laboratory Information Management System (LIMS), the data extraction and peak-identification software, data processing tools for QC and compound identification, and a collection of information interpretation and visualization tools for use by data analysts. The hardware and software foundations for these informatics components were the LAN backbone, and a database server running Oracle 10.2.0.1 Enterprise Edition.

LIMS: The purpose of the Metabolon LIMS system was to enable fully auditable laboratory automation through a secure, easy to use, and highly specialized system. The scope of the Metabolon LIMS system encompasses sample accessioning, sample preparation and instrumental analysis and reporting and advanced data analysis. All of the subsequent software systems are grounded in the LIMS data structures. It has been modified to leverage and interface with the in-house information extraction and data visualization systems, as well as third party instrumentation and data analysis software.

Data Extraction and Quality Assurance: The data extraction of the raw mass spec data files yielded information that could loaded into a relational database and manipulated without resorting to BLOB manipulation. Once in the database the information was examined and appropriate QC limits were imposed. Peaks were identified using Metabolon's proprietary peak integration software, and component parts were stored in a separate and specifically designed complex data structure.

Compound identification: Compounds were identified by comparison to library entries of purified standards or recurrent unknown entities. Identification of known chemical entities was based on comparison to metabolomic library entries of purified standards. As of this writing, more than 1000 commercially available purified standard compounds had been acquired registered into LIMS for distribution to both the LC and GC platforms for determination of their analytical characteristics. The combination of chromatographic properties and mass spectra gave an indication of a match to the specific compound or an isobaric entity. Additional entities could be identified by virtue of their recurrent nature (both chromatographic and mass spectral). These compounds have the potential to be identified by future acquisition of a matching purified standard or by classical structural analysis.

Curation: A variety of curation procedures were carried out to ensure that a high quality data set was made available for statistical analysis and data interpretation. The QC and curation processes were designed to ensure accurate and consistent identification of true chemical entities, and to remove those representing system artifacts, mis-assignments, and background noise.
Metabolon data analysts use proprietary visualization and interpretation software to confirm the consistency of peak identification among the various samples. Library matches for each compound were checked for each sample and corrected if necessary.

Normalization: For studies spanning multiple days, a data normalization step was performed to correct variation resulting from instrument inter-day tuning differences. Essentially, each compound was corrected in run-day blocks by registering the medians to equal one (1.00) and normalizing each data point proportionately (termed the "block correction"). For studies that did not require more than one day of analysis, no normalization is necessary, other than for purposes of data visualization.

Data Quality: Instrument and Process Variability

QC Sample	***Measurement***	***Median RSD***
Internal Standards	Instrument Variability	5 %
Endogenous Biochemicals	Total Process Variability	11 %

Instrument variability was determined by calculating the median relative standard deviation (RSD) for the internal standards that were added to each sample prior to injection into the mass spectrometers. Overall process variability was determined by calculating the median RSD for all endogenous metabolites (i.e., non-instrument standards) present in 100% of the Client Matrix samples, which are technical replicates of pooled client samples. Values for instrument and process variability meet Metabolon's acceptance criteria as shown in the table above.

Supplemental References:

1. Dolinay T, Kim YS, Howrylak J, Hunninghake GM, An CH, et al. (2012) Inflammasome-regulated cytokines are critical mediators of acute lung injury. Am J Respir Crit Care Med 185: 1225-1234.
2. Glickman SW, Cairns CB, Otero RM, Woods CW, Tsalik EL, et al. (2010) Disease progression in hemodynamically stable patients presenting to the emergency department with sepsis. Acad Emerg Med 17: 383-390.
3. Tsalik EL, Jones D, Nicholson B, Waring L, Liesenfeld O, et al. (2010) Multiplex PCR to diagnose bloodstream infections in patients admitted from the emergency department with sepsis. J Clin Microbiol 48: 26-33.
4. Tsalik EL, Jaggers LB, Glickman SW, Langley RJ, van Velkinburgh JC, et al. (2012) Discriminative value of inflammatory biomarkers for suspected sepsis. J Emerg Med 43: 97-106.
5. Pearl J (1988) Probabilistic reasoning in intelligent systems : networks of plausible inference. San Mateo, Calif.: Morgan Kaufmann Publishers. xix, 552 p. p.
6. Heckerman D, Gieger D (1995) Learning Bayesian Networks: A unification for discrete and Gaussian domains. Uncertainty in Artificial Intelligence: Morgan Kaufmann.
7. Chang HH, McGeachie MJ (2011) Phenotype prediction by integrative network analysis of SNP and gene expression microarrays. 33rd Annual Intl Conf of the IEEE EMBS. Boston, MA, USA. pp. 6849-6852.
8. Kass RE, Raftery AE (1995) Bayes Factors. Journal of the American Statistical Association 90: 773-795.
9. McGeachie M, Chang HH (2013) CGBayesNets: Conditional Guassian Bayesian Networks package.
10. Provost F, Fawcett T (2001) Robust Classification for Imprecise Environments. Mach Learn 42: 203-231.
11. Lasko TA, Bhagwat JG, Zou KH, Ohno-Machado L (2005) The use of receiver operating characteristic curves in biomedical informatics. J Biomed Inform 38: 404-415.
12. DeLong ER, DeLong DM, Clarke-Pearson DL (1988) Comparing the areas under two or more correlated receiver operating characteristic curves: a nonparametric approach. Biometrics 44: 837-845.
13. Langley RJ, Tsalik EL, Velkinburgh JC, Glickman SW, Rice BJ, et al. (2013) An integrated clinico-metabolomic model improves prediction of death in sepsis. Sci Transl Med 5: 195ra195.
14. Sreekumar A, Poisson LM, Rajendiran TM, Khan AP, Cao Q, et al. (2009) Metabolomic profiles delineate potential role for sarcosine in prostate cancer progression. Nature 457: 910-914.
